# Pattern recognition and cellular immune responses to novel *Mycobacterium tuberculosis-*antigens in individuals from Belarus

**DOI:** 10.1186/1471-2334-12-41

**Published:** 2012-02-15

**Authors:** Raija K Ahmed, Zoyia Rohava, Kithiganahalli N Balaji, Sven E Hoffner, Hans Gaines, Isabelle Magalhaes, Alimuddin Zumla, Alena Skrahina, Markus J Maeurer

**Affiliations:** 1 Swedish Institute for Communicable Disease Control (SMI), Solna, Sweden; 2 Republican Scientific and Practical Center for Pulmonology and Tuberculosis, Minsk, Belarus; 3 Department of Microbiology and Cell Biology, Indian Institute of Science, Bangalore, India; 4 Department of Microbiology, Tumor and Cell Biology (MTC), Karolinska Institutet, Nobelsväg 16, SE 17182 Solna, Sweden; 5 Department of Infection, University College London Medical School, Windeyer Institute of Medical Sciences, London, UK; 6 Center for allogeneic stem cell transplantation (CAST), Karolinska Hospital, Stockholm, Sweden

**Keywords:** T-cells, *M. tuberculosis*, TB, Antigen-recognition, Biomarkers

## Abstract

**Background:**

Tuberculosis (TB) is an enduring health problem worldwide and the emerging threat of multidrug resistant (MDR) TB and extensively drug resistant (XDR) TB is of particular concern. A better understanding of biomarkers associated with TB will aid to guide the development of better targets for TB diagnosis and for the development of improved TB vaccines.

**Methods:**

Recombinant proteins (n = 7) and peptide pools (n = 14) from *M. tuberculosis *(*M.tb*) antigens associated with *M.tb *pathogenicity, modification of cell lipids or cellular metabolism, were used to compare T cell immune responses defined by IFN-γ production using a whole blood assay (WBA) from i) patients with TB, ii) individuals recovered from TB and iii) individuals exposed to TB without evidence of clinical TB infection from Minsk, Belarus.

**Results:**

We identified differences in *M.tb *target peptide recognition between the test groups, i.e. a frequent recognition of antigens associated with lipid metabolism, e.g. cyclopropane fatty acyl phospholipid synthase. The pattern of peptide recognition was broader in blood from healthy individuals and those recovered from TB as compared to individuals suffering from pulmonary TB. Detection of biologically relevant *M.tb *targets was confirmed by staining for intracellular cytokines (IL-2, TNF-α and IFN-γ) in T cells from non-human primates (NHPs) after BCG vaccination.

**Conclusions:**

PBMCs from healthy individuals and those recovered from TB recognized a broader spectrum of *M.tb *antigens as compared to patients with TB. The nature of the pattern recognition of a broad panel of *M.tb *antigens will devise better strategies to identify improved diagnostics gauging previous exposure to *M.tb*; it may also guide the development of improved TB-vaccines.

## Background

Tuberculosis (TB) is one of the major global health issue, and a serious health concern in Belarus where the prevalence and spread of multidrug-resistant (MDR) TB and extensively drug resistant (XDR) TB has increased during the last few years. About 5000 individuals are newly diagnosed each year in Belarus and the prevalence of TB is 52/100,000 individuals [1]. A recent study identified MDR TB in 35.3% of newly diagnosed patients and in 76.5% of individuals who have previously been treated. XDR TB could be identified in 14.0% among the patients diagnosed with MDR TB [2]; these are alarming levels of resistant TB in Belarus.

Early diagnosis of the disease and the rapid identification of resistance to primary anti-TB drugs are essential for efficient treatment, prevention and control of TB.

The diagnosis of TB in many countries, including Belarus, still relies on the tuberculin skin test (TST) and direct sputum examination by light microscopy. The TST has low specificity due to cross-reactivity to protein purified derivative (PPD) antigens shared by environmental mycobacteria species, and may give false positive responses in Bacillus Calmette-Guerin (BCG) vaccinated individuals. BCG policies vary considerably between countries, primarily depending on the current epidemiological situation. Individuals in Belarus, as well as in Russia may be vaccinated *three times *with BCG; the pattern of cellular immune responses and antigen recognition after several BCG vaccinations has not been analyzed in detail up to now. BCG vaccination takes place early in life, BCG re-vaccination takes place during school years, a third vaccination is considered based on tuberculin skin tests (TST); BCG re-vaccination may also be postponed in adults (up to 30 years) in areas of low TB prevalence [3,4].

Interferon-γ release assays (IGRAs) have been designed to overcome the problem of cross-reactive T cell immune responses by measuring immune responses to antigens specific for *M. tuberculosis *(*M.tb*). QuantiFERON-TB Gold In-tube (QFT-GIT) [5] measures INF-γ release by sensitized T cells after stimulation with peptides from the early secreted antigenic target 6 (ESAT-6), culture filtrate protein-10 (CFP-10) and TB-7.7 which are absent in BCG and in most environmental mycobacteria [6,7]. Neither the TST nor the QTF-GIT, however, is able to discriminate between active TB-disease, latent TB-infection (LTBI) and previous TB-infection. Exposure to Mycobacteria other than tuberculosis (MOTT) may lead to false-positive results, and poor specificity of the tests may lead to unnecessary prophylactic treatment with anti-tuberculosis drugs. Thus, the ideal diagnostic test should not only discriminate LTBI from active TB, but also discriminate between TB, MOTT and previous BCG vaccination. This is of particular concern in Belarus since TB treatment can be triggered by a positive TST. In contrast, a negative TST may lead to repeated BCG vaccination according to national guidelines, since TST diameter is also used to gauge treatment responses, in addition to standard clinical and microbiological evaluation.

Although LTBI is clinically silent and not contagious, it can reactivate to cause contagious pulmonary TB [8]. Tubercle bacilli are generally considered to be non-replicating in LTBI, yet it may slowly grow and replicate [9]. LTBI is characterized by highly reduced bacterial metabolism and a significantly altered gene expression [10-12] associated with the different stages of infection [13-15]. Since most cases of active TB arise in people with LTBI, there is an urgent need to identify new potential targets for TB diagnosis and for the development of an improved TB vaccine.

One of the strategies in developing new diagnostic methods and in improving the TB vaccine involves the identification of epitopes in antigens that induce T cell responses. We have previously scanned 61 proteins from *M.tb *proteome, which facilitated the identification of potential targets to elicit T cell responses, and showed that there is a difference in peptide recognition pattern between *M.tb*-infected patients and healthy controls [16,17]. Some of these peptides were also tested for binding to the most frequent Caucasian and African MHC Class II alleles (HLA-DRB1*0101, DRB1*1501 and DRB1*0401) in different populations [17], they may serve as potential strong B and also T cell targets. The aim of this study was to compare T cell responses and INF-γ production to these candidate proteins associated with different stages of *M.tb *infection, expressed by replicating versus non-replicating bacteria.

## Methods

### Study population

The study population consists of 45 individuals from Belarus (vaccinated three times with BCG) and 17 individuals from Sweden (who did not receive BCG vaccination). The following individuals were enrolled from Belarus: i) individuals with active pulmonary TB (n = 15) were recruited from the Research Institute for Pulmonology and Tuberculosis in Minsk. TB diagnosis was based on patient history, chest X-ray and TST, confirmed by positive acid fast staining (AFS) and subsequent culture (MGIT system). The detailed characteristics of the study population are provided in the Additional file 1: Table S1. The mean age was 39 years, 60% males and 40% females ii) individuals with previous pulmonary TB (n = 15), mean age 40 years, 0% males, 100% females, all had drug-susceptible TB) and iii) clinically healthy individuals (n = 15) with no symptoms of TB at the time of sample collection (mean age 42 years, 20% males, 80% females). These 'healthy individuals' were health care workers and laboratory staff, and many of them had most likely been exposed to *M.tb*. All subjects included in this study were HIV negative. The current health care policies, diagnostic steps and TB treatment protocols have been recently reviewed and reported for Belarus [2]. Informed consent was obtained from all the subjects and the study was approved by the Local Research Ethics Committee of Belarus (No. 3, 22.07.2008). Individuals from Sweden provided informed consent; their mean age was 20 years (29% males, 71% females, immune cells analysis for *M.tb *-associated target antigens was approved by the Ethics Committee in Stockholm, Sweden; diary number 2011/863-31/2), all individuals from Sweden tested negative in the QFT-GIT test.

### TST, AFS and culture

The Mantoux skin test (TST) (Biolek, Kharkov, Ukraine) was used as a standard method for the diagnosis of TB/LTBI. The skin reaction was read after 3 days by measuring the transverse diameter of induration in mm. The classification of tuberculin reaction was: negative result = no infiltration, prick reaction 0-1 mm; doubtful result = any size of hyperemia or papula up to 5 mm; positive reaction = papule or infiltrate from 5 to 21 mm. This readout is according to the Belarus national standard criteria. Sputum was obtained from each TB-patient and acid -fast bacilli were detected by direct microscopy. The positive results in acid-fast-staining (AFS) were confirmed by culture using the BACTEC MGIT 960 (Becton Dickinson, Franklin Lakes, NJ, USA).

### QuantiFERON-TB gold in-tube (QTF-GIT)

The QFT-GIT test was performed according to the manufacturer's instructions (Cellestis, Copenhagen, Denmark). In brief, 1 ml of blood was drawn directly into vacutainer tubes, either pre-coated with saline (negative control), peptides from ESAT-6, CFP-10 and TB-7.7 or PHA (mitogen control). Tubes were incubated for 18-20 hours at 37°C in CO_2 _5% and supernatants were harvested and the amount of IFN-γ produced was measured by ELISA.

### *M.tb*-antigens

Synthetic 15-mer peptide pools were generated (JPT Peptide Technologies, Berlin, Germany) which have been found to bind to MHC class II molecules. The respective protein and peptide targets are listed in Table 1 and the individual peptide sequences are provided in the Additional file 2: Table S2. The recombinant proteins Rv3804c (Ag85A), Rv1886c (Ag85B) and Rv0288 (TB10.4) and peptide pools from Rv0959 (Ag85A/B) and Rv0288 (TB10.4) were kindly provided by the AERAS Global TB Foundation (AERAS, Washington DC, US). Recombinant ESAT-6 (Rv3875) was purchased from Statens Serum Institute (SSI, Copenhagen, Denmark). Recombinant PPE-proteins Rv0754, Rv0978c and Rv1917c [18-21] were provided by K. N. Balaji, Bangalore, India. PHA (Sigma Aldrich, Stockholm, Sweden) was used as a positive control for IFN-γ production.

**Table 1:** *M.tb *target antigens used for T cell stimulation

*M.tb *target protein	ID	Biology
Cyclopropane fatty acyl phospholipid synthase	**Rv0447c**	Involved in lipid formation and synthesis of long fatty acids, resistance to (oxidative) stress and survival

Mycobacterium bovis mycocerosic acid synthase gene (mas)	**Rv2940c**	(mas) multifunctional enzyme that catalyzes the synthesis of very long chain multiple methyl branched fatty acids called mycocerosic acids present in slow-growing pathogenic mycobacteria, major mycobacterial cell wall complex

PPE family proteins	**Rv3347c**	Accounts for about 10% of the genomic *M.tb *coding capacity. Form a source for antigenic variation among different M.tb strains
	
	**Rv1917c (p)**	Induces functional maturation of dendritic cells through an integrated cross talk between PI3K-MAPK and NF-κB signaling cascades and facilitates a Th2 phenotype

PE_PGRS 11 family proteins	**Rv0754 (p) Rv0978c (p)**	Cell wall associated protein, induces activation and maturation of human dendritic cells as well as secretion of key proinflammatory cytokines

Probable molybdopterin-guanine dinucleotide biosynthesis protein	**Rv2453c**	Involved in metabolism and respiration, associated with non replicating bacteria

Secreted antigen 85A and 85B Hypotheticall cell wall protein	**Rv3804c (85A) (p)** **Rv1886c (85B)** **(both as peptides and protein)** **Rv0959**	Mycolyl transferase activity. Responsible for the high affinity of mycobacteria to fibronectin Cell- wall associated protein

Probable lipoprotein LPRJ	**Rv1690**	Contains possible signal sequence and a prokaryotic membrane lipoprotein lipid attachment site

Secreted ESAT-6 like protein ESXH (TB10.3) ESAT-6	**Rv3019** **Rv3875 (p)**	*M.tb *pathogenicity

Possible glycosyl transferase	**Rv2957c, Rv2958c Rv2962c**	cellular metabolism and lipid formation, contributes to *M.tb *survival in macrophages

Possible hemolysin -like protein	**Rv1085c**	virulence and adaption

Probable isocitrate dehydrogenase	**Rv0066c**	cellular metabolism

Low molecular weight protein antigen 7 esxH	**Rv0288 (TB10.4) peptides and protein**	bacterial virulence

List of *M.tb *target antigens used for T-cell stimulation. Proteins are designated with (p) unless stated otherwise

### Whole blood assay (WBA)

The whole-blood assay was performed as described previously [22,23]. In brief, heparin whole blood was diluted 1:2.5 with serum-free medium (RPMI-1640 supplemented with 2 mM L-glutamine and penicillin-Streptomycin, (Invitrogen, Stockholm, Sweden). 100 μl of diluted blood was plated in duplicates in 96-well, round-bottom tissue culture plates (Nunc, Roskilde, Denmark) pre-coated with 100 μl of the diluted antigens resulting in the final volume of 200 μl in each well and the final whole blood dilution of 1:5. Peptides and proteins were used at the final concentration of 1 μg/ml and 5 μg/ml, respectively. PHA (5 μg/ml; Sigma Aldrich, Stockholm, Sweden) served as a positive control and medium diluted whole blood as a negative control. Cell cultures were incubated at 37°C with 5% CO_2_. Supernatants were harvested on day 7 and stored at -80°C until analysis. IFN-γ in cell culture supernatants was determined by a commercially available ELISA assay following the manufactures instructions (IFN-γ Eli-pair, Biosite, Stockholm, Sweden). The detection limit of the assay was 6.5 pg/ml.

### Intracellular cytokine staining (ICS)

PBMCs from non-human primates (NHP) included in our earlier vaccine studies [24] were stained as described for ICS [25]. Peripheral blood samples were obtained from female rhesus macaques (*Macaca mulatta*) of Chinese origin with an age range between 3 and 4 years. Animals were captive bred for research purposes and housed in the Astrid Fagraeus laboratory at the contained experimental facilities at the Biomedical Primate Research Centre. The local Ethical Committees approved all procedures (protocol DNR238/2006-54 and DEC#551, respectively). PBMCs were isolated from freshly obtained, heparinized peripheral blood by Ficoll-Hypaque density gradient centrifugation and processed. In brief, frozen NHP PBMCs obtained before as well as after BCG/rBCG vaccination were rested overnight and stimulated 6 h with PMA/Ionomycin (Sigma-Aldrich, Stockholm, Sweden) and peptide pools Rv0447c (putative cyclopropane fatty-acid synthase) and mixture of Rv2957 and Rv2958c (possible glycosyl transferase) in a presence of Brefeldin-A (Sigma-Aldrich, Stockholm, Sweden). Cells were stained for cell surface markers using anti-CD3-Pacific Blue (SP34-2), CD4-PerCpCy5.5 (L200) and CD8-APCCy7 (SK1). Subsequently, cells were fixed, permeabilized (IntraPrep Fix/Perm Kit, Beckman Coulter, Marseille, France) and stained with anti-IFN-γ-PECy7 (B27), TNF-α-APC and IL-2-PE (MQ1-17H12) followed by flow cytometric analysis using FACSAria flow cytometer (BD Biosciences, Stockholm, Sweden) and FlowJo software (Tree Star Inc., Ashland, OR).

### Statistical methods

The mean of the medium controls in IFN-γ ELISA plus 2 standard deviations was used as a cut-off value for the positive results. The multiplicity problem introduced by using different antigens and by comparing different groups was corrected by method for False-discovery rate (FDR). All *p*-values reported are adjusted with FDR. Fisher's exact test was used to determine the association between the outcome of each immunological measurement and different patient groups, and for comparison of the proportion of responders in each group. Statistical analyses were performed in R v2.11.0

## Results

### TST, AFS and isolation of viable bacteria

Results of TST, AFS and culture are shown in Tables 2 and 3, the detailed clinical and microbiological data for the study population with tuberculosis is provided in the Additional file 1: Table S1. A total of 43/45 (96%) of individuals tested were TST positive. Fourteen (93%) of TB patients exhibited a positive TST, including individuals with negative AFS and/or culture (Table 2), yet with typical X-ray signs. 11/15 (73%) of patients with TB tested positive for *M.tb *culture and 4/15 (27%) were positive in AFS. TST results of the individuals recovered from TB and of healthy participants are shown in Table 3. All participants from Sweden were TST negative (data not shown).

**Table 2:** Results of TST, QTF-GIT, culture and Acid-fast staining of sputum from patients from Belarus (active tuberculosis)

ID	TST (mm)	QTF-GIT	Culture	AFB
011-1	10	pos	pos	neg

011-2	16	pos	neg	neg

011-3	18	pos	pos	pos

011-4	10	pos	pos	neg

011-5	neg	neg	pos	pos

021-1	17	neg	pos	pos

021-2	9	pos	pos	neg

021-3	20	pos	pos	neg

021-4	14	neg	pos	neg

021-5	14	pos	pos	neg

021-6	14	pos	neg	neg

021-7	15	pos	neg	neg

021-8	15	pos	pos	pos

021-9	13	pos	pos	neg

021-10	12	neg	neg	neg

Total positive	14/15	11/15	11/15	4/15

**Table 3:** Results of the TST and QTF-GIT test of healthy individuals from Belarus and those recovered from TB

TB-recovered			Healthy individuals		
**ID**	**TST (mm)**	**QTF-GIT**	**ID**	**TST (mm)**	**QTF-GIT**

012-6	19	neg	013-11	9	neg

012-7	18	pos	013-12	14	pos

012-8	17	neg	013-13	12	neg

012-9	neg	neg	013-14	13	neg

012-10	15	pos	013-15	16	neg

022-1	16	neg	023-1	12	neg

022-2	17	pos	023-2	9	neg

022-3	18	pos	023-3	13	pos

022-4	17	pos	023-4	11	neg

022-5	11	pos	023-5	15	neg

022-6	15	neg	023-6	11	pos

022-7	11	neg	023-7	11	pos

022-8	15	pos	023-8	19	pos

022-9	13	pos	023-9	16	pos

022-10	14	pos	023-10	30	neg

Total positive	14/15	9/15	Total positive	15/15	6/15

### In vitro short-term IFN-γ responses to ESAT-6, CFP-10 and TB7.7

The results of the INF-γ production in response to ESAT-6, CFP-10 and TB7.7 are shown in Tables 2 and 3. 11/15 (73%) of TB+ patients, 9/15 (60%) of the individuals recovered from TB and 6/15 (40%) of healthy donors showed a positive response in a QTF-GIT test. In two TB cases, the QTF-GIT exhibited negative results, although *M.tb *could be detected by AFS and/or culture (ID 011-5 and 021-1). The cut-off for positive results was 0.35 IU/ml above the Nil (negative) control (set by the manufactures). Blood from the Swedish participants were not tested with QTF-GIT.

### In vitro long-term IFN-γ response to peptide pools

**Figure 1:**
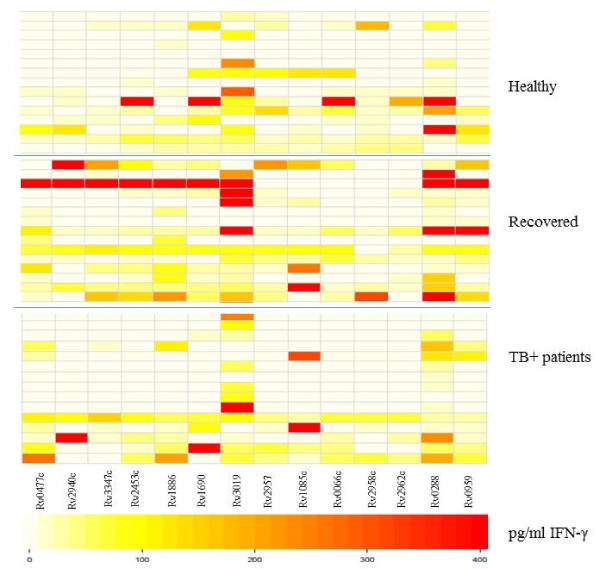
**Visualization of differences in IFN-γ production in response to *M.tb *peptide pools using heatmaps in individuals from Belarus**. Darker colors (red) represent higher and lighter colors (white) lower INF-γ production. Top panel: Healthy individuals (n = 15); Middle panel: Individuals recovered from TB (15 =); Bottom panel: TB+ patients (n = 15) from Belarus. The negative controls and constitutive IFN-γ production is subtracted.

Diluted whole-blood cultures were stimulated with the panel of *M.tb *antigens, followed by detection of IFN-γ production in culture supernatants. We identified differences in the number of responders to specific peptide pools between the groups (Figure 1). Peptides from Rv2957 elicited T cell responses in 7/15 (47%) of healthy individuals, 9/15 (60%) of individuals recovered from TB and in 4/15 (27%) of patients with TB (adjusted *p*-value = 0.39). Rv2958c was recognized by T cells in 8/15 (53%) healthy individuals followed by those recovered from TB (4/15 = 27%) and TB-patients (3/15 = 20%) (adjusted *p*-value = 0.39). 5/15 (33%) of healthy participants, 6/15 (40%) of TB-recovered and 3/15 (20%) TB-patients responded to Rv2962c (*p*-value = 0.71) (Figure 1). The non-adjusted and FDR-adjusted p-values are summarized in the Additional file 3: Table S3. Rv0447c (Cyclopropane fatty acyl phospholipid synthase) induced IFN-γ production in 11/15 (73%) individuals recovered from TB and in 7/15 (47%) TB-patients, in contrast to healthy individuals (4/15 = 27%, *p *value 0.046, after FDR adjustment: *p*-value = 0.18) (Figure 1).

Most of the individuals in each group responded to Rv3019 (ESAT-6 like protein) with the strongest IFN-γ production in the group who recovered from TB. In addition, Rv3347c peptides (PPE-family-protein) were most commonly recognized by individuals recovered from TB 8/15 (53%) (Figure 1).

Rv2453c (probable molybdopterin-guanine dinucleotide biosynthesis), associated with non-replicating bacteria, was recognized by PBMCs in 7/15 (47%) healthy individuals and in 10/15 with previous TB (67%). Only 4/15 (27%) of patients with active TB responded to this antigen (adjusted *p*-value = 0.29). The peptide antigens Rv0288 (TB10.4) and Rv0959 were recognized by most of the Belarusian participants included in the study (Figure 1).

**Figure 2:**
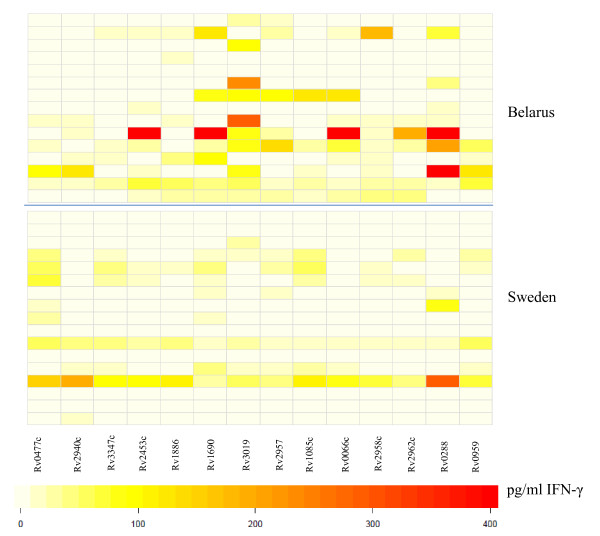
**Visualization of differences in IFN-γ production in response to *M.tb *peptide pools in PBMCs from healthy individuals from Belarus as compared to Sweden**. Darker colors (red) represent higher and lighter colors (white) lower INFγ production. Top panel: Healthy individuals from Belarus (n = 15) and Bottom panel: from Sweden (n = 15).

For comparison, we tested blood from 17 healthy, non-BCG vaccinated individuals from Sweden for T-cell reactivity directed against the identical target panel. The pattern of peptide recognition appears to be broader among participants from Belarus compared to the Swedish subjects (Figure 2). Yet, T cells from one of the Swedish participants responded to all antigens tested. Of note, several subjects from Sweden responded to Rv0447c peptides (Figure 2). There was a statistical significant difference in IFN-γ production and in the number of individuals responding to ESAT-6-like protein Rv3019 (*p *= 0.0267) from Belarus as compared to participants from Sweden.

### IFN-γ response to *M.tb *proteins

**Figure 3:**
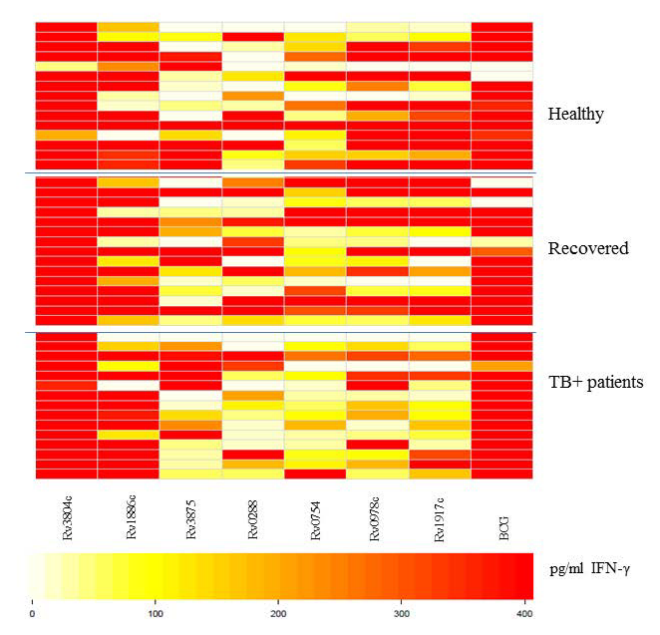
**Visualization of differences in IFN-γ production in response to *M.tb *proteins**. Blood was obtained from individuals from Belarus and incubated with different *M.tb *proteins followed by detection of IFN-γ production. Darker colors (red) represent higher and lighter colors (white) lower INF-γ production. Top panel: Healthy individuals (n = 15); Middle panel: Individuals recovered from TB (15 =). Bottom panel: TB + patients (n = 15) from Belarus.

**Figure 4:**
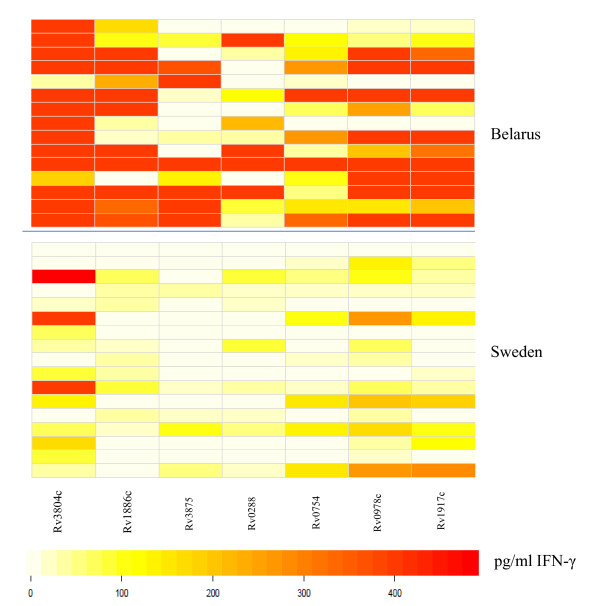
**Visualization of differences in IFN-γ production in response to proteins**. Comparison between healthy individuals from Belarus versus Sweden. Darker colors (red) represent higher and lighter colors (white) lower INF-γ production. Top panel: Healthy individuals from Belarus (n = 15) and bottom panel: from Sweden (n = 15).

A panel of *M.tb *proteins was used to gauge the cellular immune responses in blood from the cohorts in Belarus (healthy, previous TB or TB ± individuals) as well as in blood from the swedish control individuals (Figures 3 and 4). T cells from most of the individuals in all three groups from Belarus responded to the entire protein panel (Rv3804c, Rv1886c, Rv3875, Rv0288, Rv0754, Rv0978c and Rv1917) (Figure 3). There was a significant difference in INF-γ production and specific protein recognition between healthy individuals from Sweden and in healthy individuals from Belarus (Figure 4). Cells obtained from individuals from Belarus exhibited higher IFN-γ production and responded more frequently to antigens Rv3804c (*p *= 0.0170), Rv1886c (*p *= 0.0015), Rv3875 (*p *= 0.0170), Rv0754c (0.0447) and Rv1917c (0.0447) as compared to participants from Sweden. Several proteins used in this study were recognized by participants from Sweden, although the IFN-γ production was lower compared to the healthy individuals from Belarus (Figure 4).

### Candidate *M.tb *antigens are recognized by polyfunctional T cells

**Figure 5:**
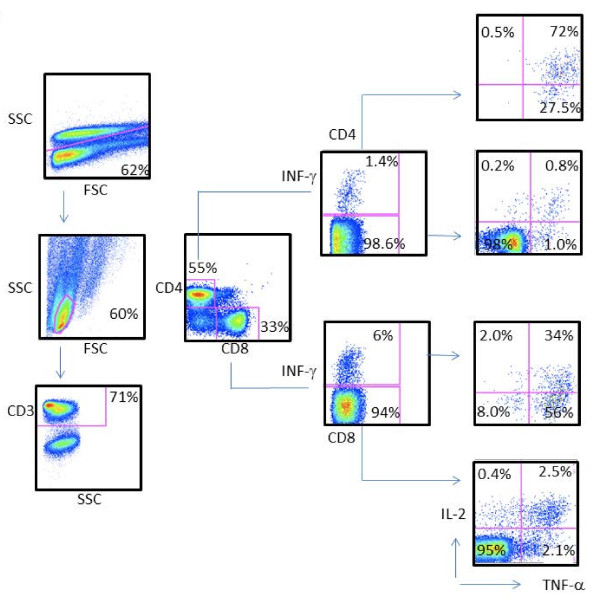
**Strong polyfunctional CD4+ and CD8+ T cell responses directed against Rv0477c *M.tb *target peptides**. PMBCs were obtained after BCG vaccination and tested by ICS for IFN-γ, TNF-α and IL-2 production in CD4+ and CD8+ T cells. First, cells were gated on site and forward scatter, followed by gating on CD3+ events and CD4+ versus CD8+ T-cells. Responding T-cells were first tested for IFN-γ production, followed by IL-2 versus TNF-α production in the IFN- γ + and IFN-γ - T cell populations. Controls included stimulation with medium or with PMA/ionomycin (data not shown).

Most the test antigens listed in Table 1 are also expressed by BCG and MOTT. At this point, we did not have access to PBMCs from individuals (i.e. infants) prior to BCG vaccination from Minsk, or as longitudinally sampled PBMCs reflecting the sequence of multiple BCG vaccinations. Although we could not control the exposure to MOTT in non-human primates (NHPs), we resorted to the analysis of more detailed cellular immune responses in PBMCs from NHP prior to and after BCG vaccination as a paradigm for testing polyfunctional T cells responding to *M.tb *test antigens. In an attempt to understand the immune responses to selected antigens seen in the study population, we performed measurements of intracellular IFN-γ, TNF-α and IL-2 production in CD4+ and CD8+ T cells from NHP in response to peptides from Rv0447c, and a cocktail from Rv2957/Rv2958c peptides using cryo-preserved PBMCs, previously obtained from NHPs included in a vaccine study [24]. As shown in Figure 5, PBMCs from NHPs vaccinated with BCG exhibited both CD4+ T cells and CD8+ T cells producing IL-2, IFN-γ and TNF-α in response to stimulation with Rv0447c (Cyclopropane fatty-acyl phospholipid synthase) and to Rv2957/Rv2958c (Glycosyltransferase). Cytokine production was higher after BCG vaccination in both CD4+ and CD8+ T cells (Additional file 4: Figure S1).

## Discussion

We used pools of several MHC class II-binding peptides from *M.tb *for their ability to stimulate IFN-γ production in vitro using whole blood from Belarusian individuals with a history of previous TB, patients with active pulmonary TB and healthy individuals (most likely TB exposed). In addition, 17 non-BCG vaccinated healthy individuals from Sweden were also included in the study. The selected peptides have shown to be expressed in replicating bacteria (Rv0477c, Rv2940c, Rv1690, Rv1085c, Rv1886, Rv2962c, Rv2958c, Rv2957c), non-replicating bacteria (Rv2453c, yet also Rv0477c, Rv2940c) and are suggested to be able to differentiate between *M.tb*, MOTT and BCG (Rv3019, Rv0066c, Rv3347c). Some peptide pools (glycosyl transferase) are more frequently recognized by T cells from healthy individuals as compared to TB+ patients suggesting that these were sensitized to mycobacteria. In addition, individuals recovered from TB recognized a broader panel of peptide pools compared to the other two groups (healthy or TB+)which indicates that the responses to *M.tb *specific antigens apparently persist in individuals who had recovered from pulmonary TB (i.e. long-term memory cellular responses). Of note, the analysis presented in the current report is based on IFN-γ production in PBMCs. The simultaneous measurement of additional chemokines/cytokines, including CXCL10 and IL-10 will most likely increase the discriminatory power and biological value of immunological readouts to *M.tb *antigens [26].

One of the strategies in developing new diagnostic methods improving TB vaccine design involves the identification of biomarkers for protection and novel T cell targets. Of particular interest is glycosyltransferase, involved in cellular metabolism and lipid formation. It has been shown that glycosyltransferase genes contribute to *M.tb *survival in macrophages [27]. Interestingly, the peptide pools from Rv2957c, Rv2958 and Rv2962c were more commonly recognized by T cells from healthy (most likely TB exposed) individuals as compared to TB-patients (Figure 1, see Additional file 3: Table S3), yet larger clinically well defined cohorts are needed in order to provide biologically relevant biomarkers to study *M.tb *immune responses and to test for novel M.tb vaccine targets.

Other peptide pools from *M.tb *proteins involved in lipid formation and synthesis of long fatty acids, Rv0477c (Cyclopropane fatty acyl phospholipid synthase) induced IFN-γ production in the majority of PBMCs from individuals suffering from TB (73%) (Figure 1). Since mycolic acids represent a major constituent of the mycobacterial cell wall complex, they provide the first line of defense against potentially lethal environmental conditions. Slow growing pathogenic mycobacteria such as *M.tb *modify their mycolic acids by cyclopropanation which is a common membrane modification in bacteria and plants [28,29] and essential for viability, drug resistance and cell wall integrity [30] playing an important role in *M.tb *pathogenesis [31,32].

Chemical inhibition of mycolic acid methyltransferases has shown to be lethal to *M.tb *and causes alterations in cell envelope structure and drug susceptibility [30]. Thus, Rv0477c may present an interesting target for development of new antibiotics against *M.tb*. Detectable cellular responses to Rv0477c, although limited, were also found in PBMCs from healthy Swedish controls. It is possible that immune recognition of this antigen is partially primed by exposure to or infection with MOTT since mycolic fatty acids are also present in MOTT. Cyclopropane fatty acids are also present in many other bacterial species expressed in stationary phase cells, including *E.coli *[29]; structural homologies may be responsible for cross-reactive T-cell responses. Of interest, a recent report showed that mutations in the rpoS-regulated genes in *E.coli *(i.e. cfa, cyclopropane fatty acid synthetase and osmB, outer membrane lipoprotein) are significantly higher sensitive to (oxidative) stress and impaired in membrane repair mechanism [33].

This notion is supported by our observation that constitutive and antigen-specific IFN-γ, IL-2 and TNFα production can be detected by ICS in CD4+ and CD8+ T-cells from NHPs prior to BCG vaccination, yet that BCG lead to a strong expansion of polyfunctional T cells. Future experiments, using T cells from animals housed under sterile conditions may show the contribution of MOTT and their role in providing an immunological matrix which can be boosted by TB vaccination strategies.

While there are no precisely defined correlates of protection against mycobacterial infection at present, IFN-γ producing Th1 cells are believed to be essential in protection against TB [34,35]. In this study we focused on INF-γ and showed that there is a difference in IFN-γ production between the study groups. At this point, we cannot determine if the IFN-γ production observed in this study was due several BCG vaccinations, due to contact with *M.tb *in Belarus, or - mutually not exclusive - to related target structures present in other bacterial species.

Rv3804c, Rv1886c and Rv0288 present in *M.tb *and BCG have been extensively used in different vaccine trials. In general, Rv3804c was most commonly recognized in each group tested. Also several Swedish subjects responded to this antigen. Furthermore, IFN-γ production was higher in response to proteins compared to the peptides (Figures 1, 2, 3, 4). Differences may be due to i) peptide instability, ii) differential antigen processing and presentation and iii) the fact that the peptide pools did not cover the entire protein antigen. These differences may also, in part, explain the strong recognition of the recombinant proteins from the PPE family members Rv0978, Rv0754 and Rv1971c in patients with TB [18-21] in PBMCs from individuals who recovered from TB and also in healthy individuals with no record with TB (yet most likely exposed, since these individuals are health care workers) (Figure 2). The same was true for target recognition from T cells obtained from individuals (TST-, non-BCG vaccinated) from Sweden (Figure 4). These antigens have been reported to provide epitopes differentiating humoral immune responses in individuals with TB. Yet more detailed experiments showed that Rv0978 and Rv0754 recognize TLR2 and induce maturation and activation of human DCs [19]. They enhance the ability of DCs to stimulate CD4+ T-cell responses and activate the ERK1/2, p38 MAPK, and NF-kappaB signaling pathways in DCs and may be instrumental to shape the quality of the innate cellular immune response in exposure to mycobacterial species. This may, in part, explain the strong IFN-γ production in response to the *M.tb *proteins; corresponding peptide cocktails may not provide these signals leading to DC maturation.

Differences in *M.tb *antigen recognition may be associated with i) the 'genetic makeup' of the test population, ii) alternate *M.tb *target molecules in circulating strains, iii) extent of TB disease, previous exposures to *M.tb *and iv) cross-reactive T-cell responses directed against closely related targets from other bacterial species: only a clinically well defined study population, along with genetic *M.tb *strain [36,37] and host immunological marker analysis will aid to visualize a more realistic pattern of anti-*M.tb *directed immune responses.

We also compared in the current study the cellular immune responses to *M.tb*-antigens in healthy individuals from Sweden and from Belarus. The number of responders to each antigen was higher and the IFN-γ production was stronger in the group of individuals from Belarus. The difference seen between the groups is most likely a reflection of the environment since Sweden is a low TB endemic country in contrast to Belarus where TB burden is high [2]. Furthermore, individuals from Belarus received two to three BCG vaccinations, which may alter the immune responses to *M.tb *proteins. None of the Swedish controls were BCG vaccinated and had no identified risk of exposure to TB.

TST is the primary method for diagnosis of latent TB infection in many countries but is not able to distinguish between BCG vaccination and reactions caused by *M.tb *infection itself. In the present study, viable *M.tb *bacilli could be detected in some patients with a positive AFS, while the immunological assays used (TST and QFT-GIT) provided negative results (Table 2). In three culture confirmed TB cases, the IGRA tested negative. All the TST positive individuals were positive for QFT-GIT within TB-positive cases. The discrepancy seen between the immunological and bacteriological assays is most likely due to not fully functional T cells from patients with TB. We did not assess the role of different HLA backgrounds in response to selected peptides in this study. Although most of the people in Belarus are Caucasian, it is still likely that some individuals may not have responded to selected target peptide pools.

## Conclusion

We show that PBMCs from healthy individuals from Belarus and those recovered from TB recognized a broader panel of *M.tb*-antigens as compared to TB-patients, defined by IFN-γ production. Individual *M.tb *antigens were strongly recognized by polyfunctional T cells in PBMCs from NHPs housed in a controlled environment after BCG vaccination. The data show that screening with selected peptide pools aids to identify biologically relevant *M.tb *targets, e.g. cyclopropane fatty-acid synthase and glycosyl transferase, which may have potential as targets for TB-diagnosis and to measure BCG vaccine take.

## Competing interests

The authors declare that they have no competing interests.

## Authors' contributions

RA was responsible for supervision and teaching of the performance of the immunological assays, data collection, analysis and wrote the manuscript, ZR was in charge of the immunology laboratory work in Belarus, KNB contributed the PE/PPE antigens and revised the manuscript, IM contributed with the NHP data, SH contributed to study design and revised the manuscript, HG was involved in the WBA set up, AZ was responsible for data interpretation and manuscript design, AS was responsible for the patient care and laboratory work in Belarus and the writing of the manuscript. MM was responsible for study design, immunological assays, drafting and writing the manuscript. All authors read and approved the final manuscript.

## Pre-publication history

The pre-publication history for this paper can be accessed here:

http://www.biomedcentral.com/1471-2334/12/41/prepub

## Supplementary Material

Additional file 1 **Table S1**. Detailed information concerning TB patients (clinic, chest-X-ray, bacteriology). Click here for file

Additional file 2 **Table S2**. Detailed listing of synthetic peptides used for T-cell reactivity testing using the wholeblood assay (WBA). Click here for file

Additional file 3 **Table S3**. P values of IFN-γ responses in PBMCs. Click here for file

Additional file 4 **Figure S1**. PBMCs from NHPs before (-) and after (+) BCG vaccination were tested for IL-2 cytokine production in CD4+ and CD8+ T-cells. Increased intracellular IL-2 production in response to Rv2947/2958 or to Rv0477c peptide stimulation in a standard 6 hr intracellular cytokine assay. Click here for file
