# Supplementary material for: Pattern recognition and cellular immune responses to novel Mycobacterium tuberculosis-antigens in individuals from Belarus
Source: BMC Infect Dis. 2012 Feb 15;12:41. doi: 10.1186/1471-2334-12-41 (PMC3305616; doi:10.1186/1471-2334-12-41)
Supplement: Additional file 1 — Table S1. Detailed information concerning TB patients (clinic, chest-X-ray, bacteriology). [file 1471-2334-12-41-S1.PDF]

Supplementary Table S1 Detailed information concerning TB patients (clinic, chest-X-ray, bacteriology)

ID	sex	Age Year of birth	TB diagnosis at study entry	bacteriology at the time of diagnosis	DST (resistance to)	Current treatment (drugs)	Chest x-ray at diagnosis	bacteriology at the time of sampling
011-1	f	1958	Bilateral pulmonary TB,<br>Pleural TB	Smear –<br>Culture +	Susceptibility to first line	Category I, Intensive phase, (H,R,E,Z)	**Bilateral upper lobes infiltrations with small cavities. Right pleural effusion**	Smear –<br>Culture +
011-2	m	1985	Upper left lobe TB	Smear –<br>Culture +	Susceptibility to first line	Category I, Intensive phase, (H,R,E,Z)	**Focal shadow in upper lobe of the left lung**	Smear –<br>Culture -
011-3	m	1951	Upper right lobe TB	Smear +<br>Culture +	Resistance to (H, R, S, E, Eto)	Category IV, Intensive phase (E,Cs,Km, Ofx, Pas)	**Several focal shadows in upper lobe of the right lung**	Smear +<br>Culture +
011-4	m	1956	Bilateral disseminated lung TB	Smear –<br>Culture +	Susceptibility to first line	Category I, Intensive phase, (H-0,3, R-0,6, E-1,6, Z-2,0)	**Bilateral various sized shadows**	Smear –<br>Culture +
011-5	m	1962	Upper right lobe TB	Smear –<br>Culture +	Susceptibility to first line	Category I, Intensive phase, (H,R,E,Z)	**Right upper lobe infiltrations with small cavities**	Smear –<br>Culture +
021-1	m	1969	Bilateral pulmonary TB	Smear +<br>Culture +	Resistance to (H, R, S, E, Eto, Km, Ofx, Am)	Category IV, Intensive phase: Cm, Cs, Eto, PAS, Amx/Clv	**Bilateral upper lobes infiltrations with cavitations**	Smear +<br>Culture +
021-2	m	1978	Upper right lobe TB	Smear –<br>Culture +	Susceptibility to first line	Category I, Intensive phase, (H,R,E,Z)	**Several focal shadows in upper lobe of the right lung**	Smear –<br>Culture +
021-3	m	1963	Upper right lobe TB	Smear –<br>Culture +	Susceptibility to first line	Category I, Intensive phase, (H,R,E,Z)	**Right upper lobe infiltration with cavitation**	Smear –<br>Culture +
021-4	m	1960	Upper left lobe TB	Smear –<br>Culture +	Susceptibility to first line	Category I, Intensive phase, (H,R,E,Z)	**Left upper lobe infiltration with several small cavitations**	Smear –<br>Culture +
021-5	m	1986	Upper right lobe TB	Smear –<br>Culture +	Susceptibility to first line	Category I, Intensive phase, (H,R,E,Z)	**Several focal shadows in upper lobe of the right lung**	Smear –<br>Culture +

021-6	f	1985	Upper right lobe TB	Smear – Culture +	Susceptibility to first line	Category I, Intensive phase, (H,R,E,Z)	**Right upper lobe infiltrations with small cavitations**	Smear – Culture +
021-7	f	1984	Upper right lobe TB	Smear – Culture +	Resistance to (H, R, S)	Category IV, Intensive phase (E, Z, Ofx, Pas, Pro, Cm)	**Several focal shadows in upper lobe of the right lung**	Smear – Culture +
021-8	f	n.a.	Upper left lobe T (relapse, TB cured in 2006)	Smear + Culture +	Resistance to (H, S, R, E, Z)	Category IV, Intensive phase (E,Z,Km,Ofx,Pas)	**Left upper lobe infiltration with several cavitations on the background of fibrotic changes**	Smear + Culture +
021-9	f	1980	Upper left lobe TB	Smear – Culture +	Resistance to (H, R, S, E, Eto)	Category IV, Intensive phase (PAS, Km, Ofx, Eto, CS)	**Left upper lobe infiltrations with several cavitations**	Smear – Culture +
021-10	m	1975	Upper right lobe TB (relapse, TB cured in 2006)	Smear – Culture +	Susceptibility to first line	Category II, Intensive phase (H, S, R, E, Z)	**Focal shadow in upper lobe of the right lung**	Smear – Culture +

f= female, m= male, n.a. not available.
