# Supplementary material for: Pattern recognition and cellular immune responses to novel Mycobacterium tuberculosis-antigens in individuals from Belarus
Source: BMC Infect Dis. 2012 Feb 15;12:41. doi: 10.1186/1471-2334-12-41 (PMC3305616; doi:10.1186/1471-2334-12-41)
Supplement: Additional file 2 — Table S2. Detailed listing of synthetic peptides used for T-cell reactivity testing using the wholeblood assay (WBA). [file 1471-2334-12-41-S2.PDF]

Ahmed et al., Supplementary Figure S1

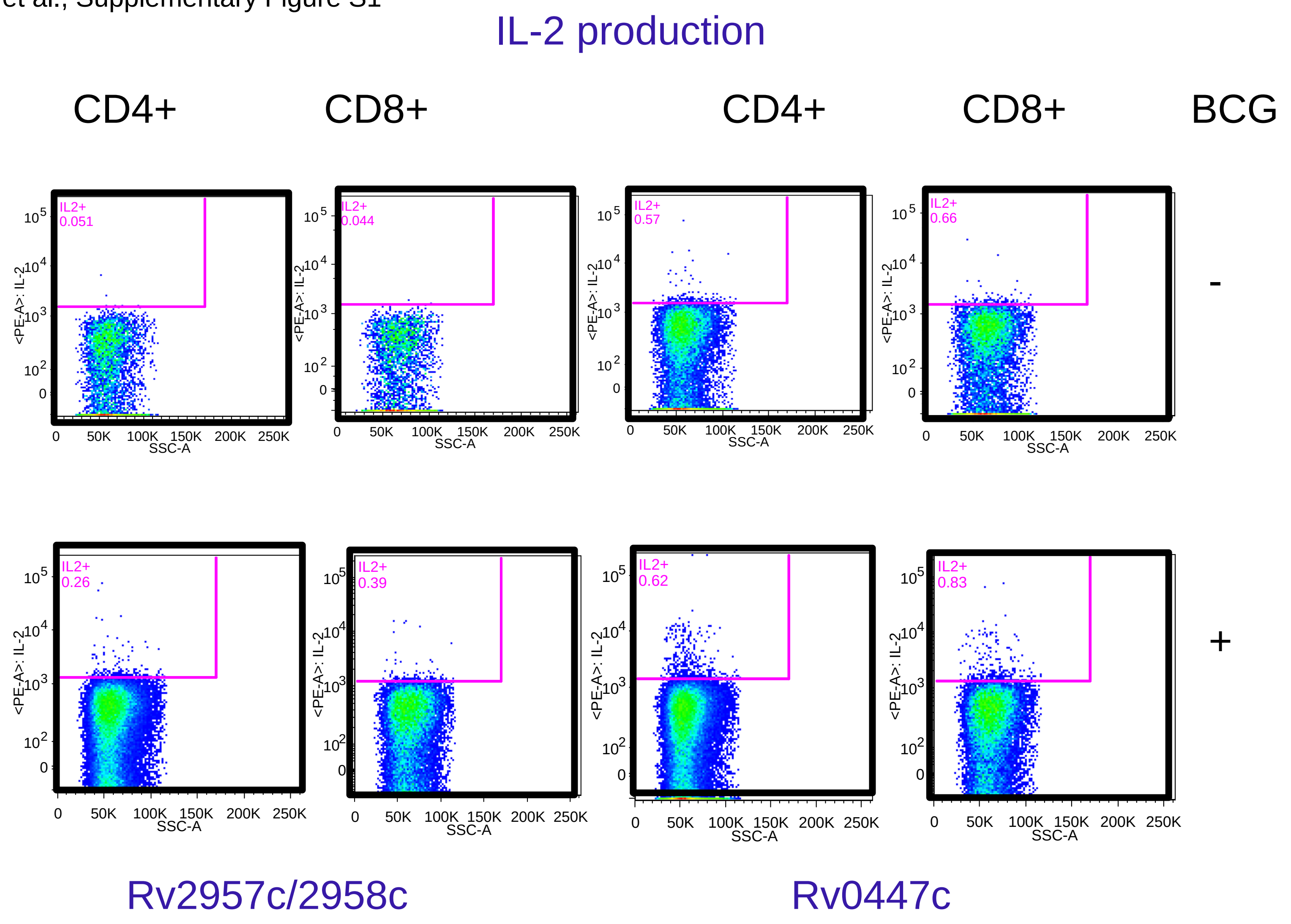

PBMCs from NHPs before (-) and after (+) BCG vaccination were tested for IL-2 cytokine production in CD4+ and CD8+ T-cells. Increased intracellular IL-2 production in response to Rv2947/2958 or to Rv0477c peptide stimulation in a standard 6hr intracellular cytokine assay.
