# Supplementary material for: Pattern recognition and cellular immune responses to novel Mycobacterium tuberculosis-antigens in individuals from Belarus
Source: BMC Infect Dis. 2012 Feb 15;12:41. doi: 10.1186/1471-2334-12-41 (PMC3305616; doi:10.1186/1471-2334-12-41)
Supplement: Additional file 3 — Table S3. P values of IFN-γ responses in PBMCs. [file 1471-2334-12-41-S3.PDF]

Ahmed et al., Supplementary Table S2

Detailed listing of synthetic peptides used for T-cell reactivity testing using the whole-blood assay (WBA).

Index-Pool	Sequence	Pool-ID	Protein
**1**	**AYSEAGFRSGYLDVY**	**Pool 1**	**PUTATIVE CYCLOPROPANE-FATTY-ACYL-PHOSPHOLIPID SYNTHASE UFAA1 NP_854118_00029**
**2**	**CAMFTDLLAQPTPAW**	**Pool 1**	**PUTATIVE CYCLOPROPANE-FATTY-ACYL-PHOSPHOLIPID SYNTHASE UFAA1 NP_854118_00029**
**3**	**EAGFRSGYLDVYQWT**	**Pool 1**	**PUTATIVE CYCLOPROPANE-FATTY-ACYL-PHOSPHOLIPID SYNTHASE UFAA1 NP_854118_00029**
**4**	**EQQRLARQRVAAAGF**	**Pool 1**	**PUTATIVE CYCLOPROPANE-FATTY-ACYL-PHOSPHOLIPID SYNTHASE UFAA1 NP_854118_00029**
**5**	**FARMWELYLAYSEAG**	**Pool 1**	**PUTATIVE CYCLOPROPANE-FATTY-ACYL-PHOSPHOLIPID SYNTHASE UFAA1 NP_854118_00029**
**6**	**FRSGYLDVYQWTLIR**	**Pool 1**	**PUTATIVE CYCLOPROPANE-FATTY-ACYL-PHOSPHOLIPID SYNTHASE UFAA1 NP_854118_00029**
**7**	**GYLDVYQWTLIREGP**	**Pool 1**	**PUTATIVE CYCLOPROPANE-FATTY-ACYL-PHOSPHOLIPID SYNTHASE UFAA1 NP_854118_00029**
**8**	**HGLIGFGESYMAGEW**	**Pool 1**	**PUTATIVE CYCLOPROPANE-FATTY-ACYL-PHOSPHOLIPID SYNTHASE UFAA1 NP_854118_00029**
**9**	**LYLAYSEAGFRSGYL**	**Pool 1**	**PUTATIVE CYCLOPROPANE-FATTY-ACYL-PHOSPHOLIPID SYNTHASE UFAA1 NP_854118_00029**
**10**	**MWELYLAYSEAGFRS**	**Pool 1**	**PUTATIVE CYCLOPROPANE-FATTY-ACYL-PHOSPHOLIPID SYNTHASE UFAA1 NP_854118_00029**
**11**	**RDQARRNIAVHYDLS**	**Pool 1**	**PUTATIVE CYCLOPROPANE-FATTY-ACYL-PHOSPHOLIPID SYNTHASE UFAA1 NP_854118_00029**
**12**	**SVTLSVEQQRLARQR**	**Pool 1**	**PUTATIVE CYCLOPROPANE-FATTY-ACYL-PHOSPHOLIPID SYNTHASE UFAA1 NP_854118_00029**
**13**	**VSVEMIEAVGYRSWP**	**Pool 1**	**PUTATIVE CYCLOPROPANE-FATTY-ACYL-PHOSPHOLIPID SYNTHASE UFAA1 NP_854118_00029**
**14**	**WIQKYIFPGGLLPST**	**Pool 1**	**PUTATIVE CYCLOPROPANE-FATTY-ACYL-PHOSPHOLIPID SYNTHASE UFAA1 NP_854118_00029**
**15**	**YLDVYQWTLIREGPP**	**Pool 1**	**PUTATIVE CYCLOPROPANE-FATTY-ACYL-PHOSPHOLIPID SYNTHASE UFAA1 NP_854118_00029**
1	ARASSRYGHWWPAVA	Pool 2	MYCOBACTERIUM BOVIS MYCOCEROSIC ACID SYNTHASE GENE; M95808_00016
2	ASVILRRTIDADRSF	Pool 2	MYCOBACTERIUM BOVIS MYCOCEROSIC ACID SYNTHASE GENE; M95808_00016
3	ATLFDPREQPVCDGA	Pool 2	MYCOBACTERIUM BOVIS MYCOCEROSIC ACID SYNTHASE GENE; M95808_00016
4	AYAFETLVRHDRAYS	Pool 2	MYCOBACTERIUM BOVIS MYCOCEROSIC ACID SYNTHASE GENE; M95808_00016
5	DETAWRNGDWYVARL	Pool 2	MYCOBACTERIUM BOVIS MYCOCEROSIC ACID SYNTHASE GENE; M95808_00016
6	DRVGGFSEGGCWRTF	Pool 2	MYCOBACTERIUM BOVIS MYCOCEROSIC ACID SYNTHASE GENE; M95808_00016
7	DTADRLVSAATATGL	Pool 2	MYCOBACTERIUM BOVIS MYCOCEROSIC ACID SYNTHASE GENE; M95808_00016
8	DVETGLFVPQAVTPW	Pool 2	MYCOBACTERIUM BOVIS MYCOCEROSIC ACID SYNTHASE GENE; M95808_00016
9	EADLDVLDEHGTVLL	Pool 2	MYCOBACTERIUM BOVIS MYCOCEROSIC ACID SYNTHASE GENE; M95808_00016
10	EEGAYAFETLVRHDR	Pool 2	MYCOBACTERIUM BOVIS MYCOCEROSIC ACID SYNTHASE GENE; M95808_00016
11	EFEGELPRLFVVTRQ	Pool 2	MYCOBACTERIUM BOVIS MYCOCEROSIC ACID SYNTHASE GENE; M95808_00016
12	EIGKADVYGNTRLGL	Pool 2	MYCOBACTERIUM BOVIS MYCOCEROSIC ACID SYNTHASE GENE; M95808_00016
13	EPGVPGRSVSRWGGF	Pool 2	MYCOBACTERIUM BOVIS MYCOCEROSIC ACID SYNTHASE GENE; M95808_00016
14	ERDRLVSERLLTLGW	Pool 2	MYCOBACTERIUM BOVIS MYCOCEROSIC ACID SYNTHASE GENE; M95808_00016
15	FAHWPRAQGLPVSAI	Pool 2	MYCOBACTERIUM BOVIS MYCOCEROSIC ACID SYNTHASE GENE; M95808_00016
16	FDPREQPVCDGAYWV	Pool 2	MYCOBACTERIUM BOVIS MYCOCEROSIC ACID SYNTHASE GENE; M95808_00016
17	FETLVRHDRAYSGYI	Pool 2	MYCOBACTERIUM BOVIS MYCOCEROSIC ACID SYNTHASE GENE; M95808_00016
18	FLAEGGEIMITPEEG	Pool 2	MYCOBACTERIUM BOVIS MYCOCEROSIC ACID SYNTHASE GENE; M95808_00016
19	GAAQRAGLELLPFGG	Pool 2	MYCOBACTERIUM BOVIS MYCOCEROSIC ACID SYNTHASE GENE; M95808_00016
20	GDPIEYRSLARVYGA	Pool 2	MYCOBACTERIUM BOVIS MYCOCEROSIC ACID SYNTHASE GENE; M95808_00016
21	GLELLPFGGRFVEIG	Pool 2	MYCOBACTERIUM BOVIS MYCOCEROSIC ACID SYNTHASE GENE; M95808_00016
22	IAPMTPKVPYYSATL	Pool 2	MYCOBACTERIUM BOVIS MYCOCEROSIC ACID SYNTHASE GENE; M95808_00016
23	IEYRSLARVYGAGTP	Pool 2	MYCOBACTERIUM BOVIS MYCOCEROSIC ACID SYNTHASE GENE; M95808_00016
24	IFAVVRGTATNQDGR	Pool 2	MYCOBACTERIUM BOVIS MYCOCEROSIC ACID SYNTHASE GENE; M95808_00016

25	ILRRTIDADRSFIEY	Pool 2	MYCOBACTERIUM BOVIS MYCOCEROSIC ACID SYNTHASE GENE; M95808_00016
26	KADVYGNTRLGLFPF	Pool 2	MYCOBACTERIUM BOVIS MYCOCEROSIC ACID SYNTHASE GENE; M95808_00016
27	LADIAPMTPKVPYYS	Pool 2	MYCOBACTERIUM BOVIS MYCOCEROSIC ACID SYNTHASE GENE; M95808_00016
28	LEKLGSQLRGRDGVV	Pool 2	MYCOBACTERIUM BOVIS MYCOCEROSIC ACID SYNTHASE GENE; M95808_00016
29	LSGSEEDETAWRNGD	Pool 2	MYCOBACTERIUM BOVIS MYCOCEROSIC ACID SYNTHASE GENE; M95808_00016
30	LVRHDRAYSGYIPIL	Pool 2	MYCOBACTERIUM BOVIS MYCOCEROSIC ACID SYNTHASE GENE; M95808_00016
31	MGSVELPAKQVNSEL	Pool 2	MYCOBACTERIUM BOVIS MYCOCEROSIC ACID SYNTHASE GENE; M95808_00016
32	MTPKVPYYSATLFDP	Pool 2	MYCOBACTERIUM BOVIS MYCOCEROSIC ACID SYNTHASE GENE; M95808_00016
33	PAVFAVQVALAATME	Pool 2	MYCOBACTERIUM BOVIS MYCOCEROSIC ACID SYNTHASE GENE; M95808_00016
34	QEQRAQGACTITVHP	Pool 2	MYCOBACTERIUM BOVIS MYCOCEROSIC ACID SYNTHASE GENE; M95808_00016
35	RAQGACTITVHPLLG	Pool 2	MYCOBACTERIUM BOVIS MYCOCEROSIC ACID SYNTHASE GENE; M95808_00016
36	SDALRQTARQLATWV	Pool 2	MYCOBACTERIUM BOVIS MYCOCEROSIC ACID SYNTHASE GENE; M95808_00016
37	SLRHGVVPPLLHFNR	Pool 2	MYCOBACTERIUM BOVIS MYCOCEROSIC ACID SYNTHASE GENE; M95808_00016
38	SLTGAAQRAGLELLP	Pool 2	MYCOBACTERIUM BOVIS MYCOCEROSIC ACID SYNTHASE GENE; M95808_00016
39	SYIITGGLGGLGLFF	Pool 2	MYCOBACTERIUM BOVIS MYCOCEROSIC ACID SYNTHASE GENE; M95808_00016
40	VQAAMEDGYRVFADV	Pool 2	MYCOBACTERIUM BOVIS MYCOCEROSIC ACID SYNTHASE GENE; M95808_00016
41	VYGNTRLGLFPFRRG	Pool 2	MYCOBACTERIUM BOVIS MYCOCEROSIC ACID SYNTHASE GENE; M95808_00016
42	WAGRLRRLLVEQASV	Pool 2	MYCOBACTERIUM BOVIS MYCOCEROSIC ACID SYNTHASE GENE; M95808_00016
43	WDADDYYDPEPGVPG	Pool 2	MYCOBACTERIUM BOVIS MYCOCEROSIC ACID SYNTHASE GENE; M95808_00016
44	YYSATLFDPREQPVC	Pool 2	MYCOBACTERIUM BOVIS MYCOCEROSIC ACID SYNTHASE GENE; M95808_00016
**1**	**AAAIAAVEAEYELMW**	**Pool 3**	**YP_177963; PPE FAMILY PROTEIN_00044**
**2**	**AFISGNFSNGVLWRG**	**Pool 3**	**YP_177963; PPE FAMILY PROTEIN_00044**
**3**	**AHFTGAFGPVVVPPI**	**Pool 3**	**YP_177963; PPE FAMILY PROTEIN_00044**
**4**	**AIVGLGTPALVSGAG**	**Pool 3**	**YP_177963; PPE FAMILY PROTEIN_00044**
**5**	**AMAAAAAPYAGWLGS**	**Pool 3**	**YP_177963; PPE FAMILY PROTEIN_00044**
**6**	**APAPPPFRLPLLFVN**	**Pool 3**	**YP_177963; PPE FAMILY PROTEIN_00044**
**7**	**DHHGLVGFSYSIEIT**	**Pool 3**	**YP_177963; PPE FAMILY PROTEIN_00044**
**8**	**DPITIFPGGFTIDPL**	**Pool 3**	**YP_177963; PPE FAMILY PROTEIN_00044**
**9**	**FGNFGANMSGWWNQA**	**Pool 3**	**YP_177963; PPE FAMILY PROTEIN_00044**
**10**	**FIVWTSSGAIGPTWY**	**Pool 3**	**YP_177963; PPE FAMILY PROTEIN_00044**
**11**	**FSNGVLWRGDYEGLW**	**Pool 3**	**YP_177963; PPE FAMILY PROTEIN_00044**
**12**	**FSTPPITIDRIPLNL**	**Pool 3**	**YP_177963; PPE FAMILY PROTEIN_00044**
**13**	**GFGNFGAGSSGWWNQ**	**Pool 3**	**YP_177963; PPE FAMILY PROTEIN_00044**
**14**	**GFSYSIEITGSTLVD**	**Pool 3**	**YP_177963; PPE FAMILY PROTEIN_00044**
**15**	**GFVTAPTQGILIHTG**	**Pool 3**	**YP_177963; PPE FAMILY PROTEIN_00044**
**16**	**GGEVSILQPFTVAPI**	**Pool 3**	**YP_177963; PPE FAMILY PROTEIN_00044**
**17**	**GGLPAFTLFPGGLNI**	**Pool 3**	**YP_177963; PPE FAMILY PROTEIN_00044**
**18**	**GILWRGNYEGLAGFS**	**Pool 3**	**YP_177963; PPE FAMILY PROTEIN_00044**
**19**	**GILWRGNYEGLFSYS**	**Pool 3**	**YP_177963; PPE FAMILY PROTEIN_00044**
**20**	**GLAGFSFGYPIPLFP**	**Pool 3**	**YP_177963; PPE FAMILY PROTEIN_00044**
**21**	**GLFSYSYSLDVPRIT**	**Pool 3**	**YP_177963; PPE FAMILY PROTEIN_00044**
**22**	**GVLWRGDYEGLWGLS**	**Pool 3**	**YP_177963; PPE FAMILY PROTEIN_00044**
**23**	**GVSGFGNFGAGSSGW**	**Pool 3**	**YP_177963; PPE FAMILY PROTEIN_00044**
**24**	**HETMVIPPIVFLPSM**	**Pool 3**	**YP_177963; PPE FAMILY PROTEIN_00044**
**25**	**IDAITLFPGGLTFPA**	**Pool 3**	**YP_177963; PPE FAMILY PROTEIN_00044**
**26**	**IEPFIVWTSSGAIGP**	**Pool 3**	**YP_177963; PPE FAMILY PROTEIN_00044**

**27**	**IGPTWYSVGRIYNAG**	**Pool 3**	**YP_177963; PPE FAMILY PROTEIN_00044**
**28**	**INSVLMYSGAGSSPL**	**Pool 3**	**YP_177963; PPE FAMILY PROTEIN_00044**
**29**	**IPAFGGGTAIPISVG**	**Pool 3**	**YP_177963; PPE FAMILY PROTEIN_00044**
**30**	**ITLFPAQNFNTTFPV**	**Pool 3**	**YP_177963; PPE FAMILY PROTEIN_00044**
**31**	**ITLFPGGLTFPANSL**	**Pool 3**	**YP_177963; PPE FAMILY PROTEIN_00044**
**32**	**LNIHQTFSLGPLVVP**	**Pool 3**	**YP_177963; PPE FAMILY PROTEIN_00044**
**33**	**MVIPPIVFLPSMTIG**	**Pool 3**	**YP_177963; PPE FAMILY PROTEIN_00044**
**34**	**NADGELYVIAGDIPL**	**Pool 3**	**YP_177963; PPE FAMILY PROTEIN_00044**
**35**	**NYEGLAGFSFGYPIP**	**Pool 3**	**YP_177963; PPE FAMILY PROTEIN_00044**
**36**	**NYEGLFSYSYSLDVP**	**Pool 3**	**YP_177963; PPE FAMILY PROTEIN_00044**
**37**	**PGSIDAITLFPGGLT**	**Pool 3**	**YP_177963; PPE FAMILY PROTEIN_00044**
**38**	**PIQILPTIPLNIHQT**	**Pool 3**	**YP_177963; PPE FAMILY PROTEIN_00044**
**39**	**PITISPITLFPAQNF**	**Pool 3**	**YP_177963; PPE FAMILY PROTEIN_00044**
**40**	**PPIVFLPSMTIGGQT**	**Pool 3**	**YP_177963; PPE FAMILY PROTEIN_00044**
**41**	**PPPFRLPLLFVNALG**	**Pool 3**	**YP_177963; PPE FAMILY PROTEIN_00044**
**42**	**PVLPPEINSVLMYSG**	**Pool 3**	**YP_177963; PPE FAMILY PROTEIN_00044**
**43**	**RPSFSFFAVGPDGMP**	**Pool 3**	**YP_177963; PPE FAMILY PROTEIN_00044**
**44**	**SGFGNFGAGSSGWWN**	**Pool 3**	**YP_177963; PPE FAMILY PROTEIN_00044**
**45**	**SGNFSNGVLWRGDYE**	**Pool 3**	**YP_177963; PPE FAMILY PROTEIN_00044**
**46**	**SNVQLNPFNVNLKLQ**	**Pool 3**	**YP_177963; PPE FAMILY PROTEIN_00044**
**47**	**TGNFSNGILWRGNYE**	**Pool 3**	**YP_177963; PPE FAMILY PROTEIN_00044**
**48**	**TIFPGGFTIDPLPLS**	**Pool 3**	**YP_177963; PPE FAMILY PROTEIN_00044**
**49**	**TSEIEPFIVWTSSGA**	**Pool 3**	**YP_177963; PPE FAMILY PROTEIN_00044**
**50**	**TVLPIQILPTIPLNI**	**Pool 3**	**YP_177963; PPE FAMILY PROTEIN_00044**
**51**	**TWYSVGRIYNAGDLF**	**Pool 3**	**YP_177963; PPE FAMILY PROTEIN_00044**
**52**	**VFLPSMTIGGQTYTI**	**Pool 3**	**YP_177963; PPE FAMILY PROTEIN_00044**
**53**	**VGATSEIEPFIVWTS**	**Pool 3**	**YP_177963; PPE FAMILY PROTEIN_00044**
**54**	**VSLPIFGFGGAPGFG**	**Pool 3**	**YP_177963; PPE FAMILY PROTEIN_00044**
**55**	**VTIPTITTSPIPLKI**	**Pool 3**	**YP_177963; PPE FAMILY PROTEIN_00044**
**56**	**WRGNYEGLAGFSFGY**	**Pool 3**	**YP_177963; PPE FAMILY PROTEIN_00044**
**57**	**WRGNYEGLFSYSYSL**	**Pool 3**	**YP_177963; PPE FAMILY PROTEIN_00044**
**58**	**WSTPAVTIFPNGISI**	**Pool 3**	**YP_177963; PPE FAMILY PROTEIN_00044**
**59**	**WTGDHHGLVGFSYSI**	**Pool 3**	**YP_177963; PPE FAMILY PROTEIN_00044**
**60**	**WTSSGAIGPTWYSVG**	**Pool 3**	**YP_177963; PPE FAMILY PROTEIN_00044**
**61**	**YNTAIVGLGTPALVS**	**Pool 3**	**YP_177963; PPE FAMILY PROTEIN_00044**
1	DALRIVMADSRPLTN	Pool 4	gi;2791493;emb;CAA16030.1; PROBABLE MOLYBDOPTERIN-GUANINE DINUCLEOTIDE BIOSYNTHESIS PROTEIN _00015
2	GRNHYLAAVYRTDLA	Pool 4	gi;2791493;emb;CAA16030.1; PROBABLE MOLYBDOPTERIN-GUANINE DINUCLEOTIDE BIOSYNTHESIS PROTEIN _00015
3	GVRLAFVCAVDMPYL	Pool 4	gi;2791493;emb;CAA16030.1; PROBABLE MOLYBDOPTERIN-GUANINE DINUCLEOTIDE BIOSYNTHESIS PROTEIN _00015
4	HMVGILGQRCAPVFV	Pool 4	gi;2791493;emb;CAA16030.1; PROBABLE MOLYBDOPTERIN-GUANINE DINUCLEOTIDE BIOSYNTHESIS PROTEIN _00015
5	HYLAAVYRTDLADRV	Pool 4	gi;2791493;emb;CAA16030.1; PROBABLE MOLYBDOPTERIN-GUANINE DINUCLEOTIDE BIOSYNTHESIS PROTEIN _00015
6	VVLPWDGRNHYLAAV	Pool 4	gi;2791493;emb;CAA16030.1; PROBABLE MOLYBDOPTERIN-GUANINE DINUCLEOTIDE BIOSYNTHESIS PROTEIN _00015
**1**	**AFEWYYQSGLSIVMP**	**Pool 5**	**gi;2225974;emb;CAB10044.1; SECRETED ANTIGEN 85-B FBPB (85B) _00026**
**2**	**AGCQTYKWETFLTSE**	**Pool 5**	**gi;2225974;emb;CAB10044.1; SECRETED ANTIGEN 85-B FBPB (85B) _00026**
**3**	**AMILAAYHPQQFIYA**	**Pool 5**	**gi;2225974;emb;CAB10044.1; SECRETED ANTIGEN 85-B FBPB (85B) _00026**
**4**	**AVFNFPPNGTHSWEY**	**Pool 5**	**gi;2225974;emb;CAB10044.1; SECRETED ANTIGEN 85-B FBPB (85B) _00026**
**5**	**FLENFVRSSNLKFQD**	**Pool 5**	**gi;2225974;emb;CAB10044.1; SECRETED ANTIGEN 85-B FBPB (85B) _00026**

**6**	**GSSAMILAAYHPQQF**	**Pool 5**	**gi;2225974;emb;CAB10044.1; SECRETED ANTIGEN 85-B FBPB (85B) _00026**
**7**	**LAAYHPQQFIYAGSL**	**Pool 5**	**gi;2225974;emb;CAB10044.1; SECRETED ANTIGEN 85-B FBPB (85B) _00026**
**8**	**NFPPNGTHSWEYWGA**	**Pool 5**	**gi;2225974;emb;CAB10044.1; SECRETED ANTIGEN 85-B FBPB (85B) _00026**
**9**	**NFVRSSNLKFQDAYN**	**Pool 5**	**gi;2225974;emb;CAB10044.1; SECRETED ANTIGEN 85-B FBPB (85B) _00026**
**10**	**NTPAFEWYYQSGLSI**	**Pool 5**	**gi;2225974;emb;CAB10044.1; SECRETED ANTIGEN 85-B FBPB (85B) _00026**
**11**	**NTRLWVYCGNGTPNE**	**Pool 5**	**gi;2225974;emb;CAB10044.1; SECRETED ANTIGEN 85-B FBPB (85B) _00026**
**12**	**QDDYNGWDINTPAFE**	**Pool 5**	**gi;2225974;emb;CAB10044.1; SECRETED ANTIGEN 85-B FBPB (85B) _00026**
**13**	**QSSFYSDWYSPACGK**	**Pool 5**	**gi;2225974;emb;CAB10044.1; SECRETED ANTIGEN 85-B FBPB (85B) _00026**
**14**	**SMAGSSAMILAAYHP**	**Pool 5**	**gi;2225974;emb;CAB10044.1; SECRETED ANTIGEN 85-B FBPB (85B) _00026**
**15**	**THSWEYWGAQLNAMK**	**Pool 5**	**gi;2225974;emb;CAB10044.1; SECRETED ANTIGEN 85-B FBPB (85B) _00026**
**16**	**VANNTRLWVYCGNGT**	**Pool 5**	**gi;2225974;emb;CAB10044.1; SECRETED ANTIGEN 85-B FBPB (85B) _00026**
**17**	**VGGQSSFYSDWYSPA**	**Pool 5**	**gi;2225974;emb;CAB10044.1; SECRETED ANTIGEN 85-B FBPB (85B) _00026**
**18**	**VMPVGGQSSFYSDWY**	**Pool 5**	**gi;2225974;emb;CAB10044.1; SECRETED ANTIGEN 85-B FBPB (85B) _00026**
**19**	**VVAMAAIATFAAPVA**	**Pool 5**	**gi;2225974;emb;CAB10044.1; SECRETED ANTIGEN 85-B FBPB (85B) _00026**
**20**	**VYLLDGLRAQDDYNG**	**Pool 5**	**gi;2225974;emb;CAB10044.1; SECRETED ANTIGEN 85-B FBPB (85B) _00026**
**21**	**WDINTPAFEWYYQSG**	**Pool 5**	**gi;2225974;emb;CAB10044.1; SECRETED ANTIGEN 85-B FBPB (85B) _00026**
**22**	**WYYQSGLSIVMPVGG**	**Pool 5**	**gi;2225974;emb;CAB10044.1; SECRETED ANTIGEN 85-B FBPB (85B) _00026**
**23**	**YHPQQFIYAGSLSAL**	**Pool 5**	**gi;2225974;emb;CAB10044.1; SECRETED ANTIGEN 85-B FBPB (85B) _00026**
**24**	**YNGWDINTPAFEWYY**	**Pool 5**	**gi;2225974;emb;CAB10044.1; SECRETED ANTIGEN 85-B FBPB (85B) _00026**
1	CPAVIAPLMPNRLQA	Pool 6	gi;2326733;emb;CAB10947.1; PROBABLE LIPOPROTEIN LPRJ_00016
2	MASTFTIVAIGTYCP	Pool 6	gi;2326733;emb;CAB10947.1; PROBABLE LIPOPROTEIN LPRJ_00016
3	MMGNAFLTALTNAGI	Pool 6	gi;2326733;emb;CAB10947.1; PROBABLE LIPOPROTEIN LPRJ_00016
4	NAFLTALTNAGIAYD	Pool 6	gi;2326733;emb;CAB10947.1; PROBABLE LIPOPROTEIN LPRJ_00016
5	NGMSRDMASTFTIVA	Pool 6	gi;2326733;emb;CAB10947.1; PROBABLE LIPOPROTEIN LPRJ_00016
6	TFTIVAIGTYCPAVI	Pool 6	gi;2326733;emb;CAB10947.1; PROBABLE LIPOPROTEIN LPRJ_00016
**1**	**AVLSSAWQGDTGITY**	**Pool 7**	**gi;2791616;emb;CAA16104.1; SECRETED ESAT-6 LIKE PROTEIN ESXR_00013**
**2**	**DIASEQAVLSSAWQG**	**Pool 7**	**gi;2791616;emb;CAA16104.1; SECRETED ESAT-6 LIKE PROTEIN ESXR_00013**
**3**	**DTGITYQGWQTQWNQ**	**Pool 7**	**gi;2791616;emb;CAA16104.1; SECRETED ESAT-6 LIKE PROTEIN ESXR_00013**
**4**	**ITYQGWQTQWNQALE**	**Pool 7**	**gi;2791616;emb;CAA16104.1; SECRETED ESAT-6 LIKE PROTEIN ESXR_00013**
**5**	**SMYSYPAMTANVGDM**	**Pool 7**	**gi;2791616;emb;CAA16104.1; SECRETED ESAT-6 LIKE PROTEIN ESXR_00013**
**6**	**SSAWQGDTGITYQGW**	**Pool 7**	**gi;2791616;emb;CAA16104.1; SECRETED ESAT-6 LIKE PROTEIN ESXR_00013**
**7**	**WQGDTGITYQGWQTQ**	**Pool 7**	**gi;2791616;emb;CAA16104.1; SECRETED ESAT-6 LIKE PROTEIN ESXR_00013**
1	DWDFNIRCFSNPALV	Pool 8	POSSIBLE GLYCOSYL TRANSFERASE CAB05419_00061
2	GIRLVIVLVRRWPKV	Pool 8	POSSIBLE GLYCOSYL TRANSFERASE CAB05419_00061
3	IGPYNLRYRVLADWD	Pool 8	POSSIBLE GLYCOSYL TRANSFERASE CAB05419_00061
4	YNLRYRVLADWDFNI	Pool 8	POSSIBLE GLYCOSYL TRANSFERASE CAB05419_00061
**1**	**AYTAATITMFTVSAT**	**Pool 9**	**POSSIBLE HEMOLYSIN-LIKE PROTEIN CAA17201_00054**
**2**	**CWPAAPRSVGVPLYL**	**Pool 9**	**POSSIBLE HEMOLYSIN-LIKE PROTEIN CAA17201_00054**
**3**	**GGALYSIGGILYAVR**	**Pool 9**	**POSSIBLE HEMOLYSIN-LIKE PROTEIN CAA17201_00054**
**4**	**GVPLYLLLGWVAVWY**	**Pool 9**	**POSSIBLE HEMOLYSIN-LIKE PROTEIN CAA17201_00054**
**5**	**IGGILYAVRWPDPWP**	**Pool 9**	**POSSIBLE HEMOLYSIN-LIKE PROTEIN CAA17201_00054**
**6**	**ILYAVRWPDPWPTTF**	**Pool 9**	**POSSIBLE HEMOLYSIN-LIKE PROTEIN CAA17201_00054**
**7**	**LFVGGALYSIGGILY**	**Pool 9**	**POSSIBLE HEMOLYSIN-LIKE PROTEIN CAA17201_00054**
**8**	**LYLLLGWVAVWYTAT**	**Pool 9**	**POSSIBLE HEMOLYSIN-LIKE PROTEIN CAA17201_00054**
**9**	**LYSIGGILYAVRWPD**	**Pool 9**	**POSSIBLE HEMOLYSIN-LIKE PROTEIN CAA17201_00054**
**10**	**MIFVFIAGSYTPFAL**	**Pool 9**	**POSSIBLE HEMOLYSIN-LIKE PROTEIN CAA17201_00054**

**11**	**VWYTATILHNAGVTA**	**Pool 9**	**POSSIBLE HEMOLYSIN-LIKE PROTEIN CAA17201_00054**
**12**	**WPDPWPTTFGYHEFF**	**Pool 9**	**POSSIBLE HEMOLYSIN-LIKE PROTEIN CAA17201_00054**
**13**	**WVAVWYTATILHNAG**	**Pool 9**	**POSSIBLE HEMOLYSIN-LIKE PROTEIN CAA17201_00054**
1	APLLATYAFLPIVRA	Pool 10	PROBABLE ISOCITRATE DEHYDROGENASE CAA16247_00001
2	CDFYEEQMQDAFETG	Pool 10	PROBABLE ISOCITRATE DEHYDROGENASE CAA16247_00001
3	CERLVRGLDTIAATG	Pool 10	PROBABLE ISOCITRATE DEHYDROGENASE CAA16247_00001
4	DAFETGVMFSLHVKA	Pool 10	PROBABLE ISOCITRATE DEHYDROGENASE CAA16247_00001
5	DDLGVNVNNGLSDLY	Pool 10	PROBABLE ISOCITRATE DEHYDROGENASE CAA16247_00001
6	DISVAARILAEFPDY	Pool 10	PROBABLE ISOCITRATE DEHYDROGENASE CAA16247_00001
7	DNRGSQFYLAMYWAQ	Pool 10	PROBABLE ISOCITRATE DEHYDROGENASE CAA16247_00001
8	DTNIIKLPNISASVP	Pool 10	PROBABLE ISOCITRATE DEHYDROGENASE CAA16247_00001
9	EASDISVAARILAEF	Pool 10	PROBABLE ISOCITRATE DEHYDROGENASE CAA16247_00001
10	EQPTIIYTLTDEAPL	Pool 10	PROBABLE ISOCITRATE DEHYDROGENASE CAA16247_00001
11	GEVLLTENVEAGDIW	Pool 10	PROBABLE ISOCITRATE DEHYDROGENASE CAA16247_00001
12	GMPVLFWLDPYRPHE	Pool 10	PROBABLE ISOCITRATE DEHYDROGENASE CAA16247_00001
13	HPIVFGHAVRIFYKD	Pool 10	PROBABLE ISOCITRATE DEHYDROGENASE CAA16247_00001
14	IIKLPNISASVPQLV	Pool 10	PROBABLE ISOCITRATE DEHYDROGENASE CAA16247_00001
15	IVDVATGEVLLTENV	Pool 10	PROBABLE ISOCITRATE DEHYDROGENASE CAA16247_00001
16	KALCDFYEEQMQDAF	Pool 10	PROBABLE ISOCITRATE DEHYDROGENASE CAA16247_00001
17	KVSHPIVFGHAVRIF	Pool 10	PROBABLE ISOCITRATE DEHYDROGENASE CAA16247_00001
18	LATYAFLPIVRAFAE	Pool 10	PROBABLE ISOCITRATE DEHYDROGENASE CAA16247_00001
19	LLAKSGKTIVLKPEV	Pool 10	PROBABLE ISOCITRATE DEHYDROGENASE CAA16247_00001
20	LPIVRAFAEPAGIKI	Pool 10	PROBABLE ISOCITRATE DEHYDROGENASE CAA16247_00001
21	MSAEQPTIIYTLTDE	Pool 10	PROBABLE ISOCITRATE DEHYDROGENASE CAA16247_00001
22	QGEPVDIGGYYAPDS	Pool 10	PROBABLE ISOCITRATE DEHYDROGENASE CAA16247_00001
23	TDEAPLLATYAFLPI	Pool 10	PROBABLE ISOCITRATE DEHYDROGENASE CAA16247_00001
24	TEVQGEPVDIGGYYA	Pool 10	PROBABLE ISOCITRATE DEHYDROGENASE CAA16247_00001
25	VAARILAEFPDYLTE	Pool 10	PROBABLE ISOCITRATE DEHYDROGENASE CAA16247_00001
26	VANIVDVATGEVLLT	Pool 10	PROBABLE ISOCITRATE DEHYDROGENASE CAA16247_00001
27	VFGHAVRIFYKDAFA	Pool 10	PROBABLE ISOCITRATE DEHYDROGENASE CAA16247_00001
28	VIDSMFMSKKALCDF	Pool 10	PROBABLE ISOCITRATE DEHYDROGENASE CAA16247_00001
29	VLFWLDPYRPHENEL	Pool 10	PROBABLE ISOCITRATE DEHYDROGENASE CAA16247_00001
30	WDSLGEFLALGAGFE	Pool 10	PROBABLE ISOCITRATE DEHYDROGENASE CAA16247_00001
31	YAFLPIVRAFAEPAG	Pool 10	PROBABLE ISOCITRATE DEHYDROGENASE CAA16247_00001
32	YQEIINFCKTNGQFD	Pool 10	PROBABLE ISOCITRATE DEHYDROGENASE CAA16247_00001
**1**	**AGIPYIAIANAYWSP**	**Pool 11**	**POSSIBLE GLYCOSYL TRANSFERASE CAB05418_00023**
**2**	**AHVVRPFVLARSLDP**	**Pool 11**	**POSSIBLE GLYCOSYL TRANSFERASE CAB05418_00023**
**3**	**ARLAGIPYIAIANAY**	**Pool 11**	**POSSIBLE GLYCOSYL TRANSFERASE CAB05418_00023**
**4**	**DNRLSLSVSARLAGI**	**Pool 11**	**POSSIBLE GLYCOSYL TRANSFERASE CAB05418_00023**
**5**	**DPRFNKLLGPLPFPH**	**Pool 11**	**POSSIBLE GLYCOSYL TRANSFERASE CAB05418_00023**
**6**	**EVLLKIAQGRLFYNT**	**Pool 11**	**POSSIBLE GLYCOSYL TRANSFERASE CAB05418_00023**
**7**	**FTDGDYTLYADVPEL**	**Pool 11**	**POSSIBLE GLYCOSYL TRANSFERASE CAB05418_00023**
**8**	**FVLARSLDPSRYEVH**	**Pool 11**	**POSSIBLE GLYCOSYL TRANSFERASE CAB05418_00023**
**9**	**LKIAQGRLFYNTRTL**	**Pool 11**	**POSSIBLE GLYCOSYL TRANSFERASE CAB05418_00023**
**10**	**LPTDRPIIYATLGSS**	**Pool 11**	**POSSIBLE GLYCOSYL TRANSFERASE CAB05418_00023**
**11**	**LPVTVIAATAGRNHL**	**Pool 11**	**POSSIBLE GLYCOSYL TRANSFERASE CAB05418_00023**

**12**	**PANHRYLGPVLWSPD**	**Pool 11**	**POSSIBLE GLYCOSYL TRANSFERASE CAB05418_00023**
**13**	**PTWWHSLPTDRPIIY**	**Pool 11**	**POSSIBLE GLYCOSYL TRANSFERASE CAB05418_00023**
**14**	**PYIAIANAYWSPQAR**	**Pool 11**	**POSSIBLE GLYCOSYL TRANSFERASE CAB05418_00023**
**15**	**RKYIAADRKILNEIA**	**Pool 11**	**POSSIBLE GLYCOSYL TRANSFERASE CAB05418_00023**
**16**	**SVSARLAGIPYIAIA**	**Pool 11**	**POSSIBLE GLYCOSYL TRANSFERASE CAB05418_00023**
**17**	**WDLCRIFTDGDYTLY**	**Pool 11**	**POSSIBLE GLYCOSYL TRANSFERASE CAB05418_00023**
1	AEAVTLAHVVRPFAL	Pool 12	POSSIBLE GLYCOSYL TRANSFERASE CAB05415_00173
2	ANHEYLGPVLWSPAG	Pool 12	POSSIBLE GLYCOSYL TRANSFERASE CAB05415_00173
3	AQRRFPLPDVIWTRL	Pool 12	POSSIBLE GLYCOSYL TRANSFERASE CAB05415_00173
4	AVERAGAGVLLRTER	Pool 12	POSSIBLE GLYCOSYL TRANSFERASE CAB05415_00173
5	DHTLYADVPELMPTY	Pool 12	POSSIBLE GLYCOSYL TRANSFERASE CAB05415_00173
6	DPRYNQLLGPLPFRH	Pool 12	POSSIBLE GLYCOSYL TRANSFERASE CAB05415_00173
7	ERPLLFALQCMPLNW	Pool 12	POSSIBLE GLYCOSYL TRANSFERASE CAB05415_00173
8	FGVRLVKLLYRLERP	Pool 12	POSSIBLE GLYCOSYL TRANSFERASE CAB05415_00173
9	GIPYIAIANAYWSPY	Pool 12	POSSIBLE GLYCOSYL TRANSFERASE CAB05415_00173
10	LDPSRYEVHFACDPR	Pool 12	POSSIBLE GLYCOSYL TRANSFERASE CAB05415_00173
11	LLRTERLKSQRVAGA	Pool 12	POSSIBLE GLYCOSYL TRANSFERASE CAB05415_00173
12	LYADVPELMPTYDLP	Pool 12	POSSIBLE GLYCOSYL TRANSFERASE CAB05415_00173
13	PSERFFGNLTQGRFY	Pool 12	POSSIBLE GLYCOSYL TRANSFERASE CAB05415_00173
14	RAGAGVLLRTERLKS	Pool 12	POSSIBLE GLYCOSYL TRANSFERASE CAB05415_00173
15	RLAGIPYIAIANAYW	Pool 12	POSSIBLE GLYCOSYL TRANSFERASE CAB05415_00173
16	SPYAQRRFPLPDVIW	Pool 12	POSSIBLE GLYCOSYL TRANSFERASE CAB05415_00173
17	TWWDSLPTDRPIVYA	Pool 12	POSSIBLE GLYCOSYL TRANSFERASE CAB05415_00173
18	VSARLAGIPYIAIAN	Pool 12	POSSIBLE GLYCOSYL TRANSFERASE CAB05415_00173
19	YIAIANAYWSPYAQR	Pool 12	POSSIBLE GLYCOSYL TRANSFERASE CAB05415_00173
20	YNQLLGPLPFRHHAI	Pool 12	POSSIBLE GLYCOSYL TRANSFERASE CAB05415_00173
