# Supplementary material for: Pattern recognition and cellular immune responses to novel Mycobacterium tuberculosis-antigens in individuals from Belarus
Source: BMC Infect Dis. 2012 Feb 15;12:41. doi: 10.1186/1471-2334-12-41 (PMC3305616; doi:10.1186/1471-2334-12-41)
Supplement: Additional file 4 — Figure S1. PBMCs from NHPs before (-) and after (+) BCG vaccination were tested for IL-2 cytokine production in CD4+ and CD8+ T-cells. Increased intracellular IL-2 production in response to Rv2947/2958 or to Rv0477c peptide stimulation in a standard 6 hr intracellular cytokine assay. [file 1471-2334-12-41-S4.PDF]

Supplementary Table S3

P values of IFN-gamma responses in PBMCs

PEPTIDE POOLS

TARGET	H	P	TB	H1	P1	TB1	p.value	p.value.adj
Rv0447c	4 (27)	11 (73)	7 (47)	4	11	7	0.0462158941526575	0.189573053595724
Rv2940c	5 (33)	6 (40)	2 (13)	5	6	2	0.345997290030908	0.484396206043272
Rv3347c	4 (27)	8 (53)	4 (27)	4	8	4	0.251226232008674	0.390796360902382
Rv2453c	7 (47)	10 (67)	4 (27)	7	10	4	0.105889381785584	0.296490268999636
Rv1886	6 (40)	12 (80)	6 (40)	6	12	6	0.0541637295987783	0.189573053595724
Rv1690	7 (47)	9 (60)	4 (27)	7	9	4	0.220036423762817	0.38506374158493
Rv3019	10 (67)	11 (73)	11 (73)	10	11	11	1	1
Rv2957	7 (47)	9 (60)	4 (27)	7	9	4	0.220036423762817	0.38506374158493
Rv1085c	5 (33)	7 (47)	5 (33)	5	7	5	0.79541102481827	0.85659648826583
Rv0066c	8 (53)	6 (40)	4 (27)	8	6	4	0.388450855818421	0.494391998314355
Rv2958c	8 (53)	4 (27)	3 (20)	8	4	3	0.208115701810516	0.38506374158493
Rv2962	5 (33)	6 (40)	3 (20)	5	6	3	0.611047216434401	0.712888419173468
Rv0959	4 (27)	11 (73)	7 (47)	4	11	7	0.0462158941526575	0.189573053595724
Rv0288	8 (53)	14 (93)	12 (80)	8	14	12	0.0484287015618176	0.189573053595724

RECOMBINANT PROTEINS

TARGET	H	P	TB	H1	P1	TB1	p.value	p.value.adj
Rv0288 (TB10.4)	11 (73)	14 (93)	13 (87)	11	14	13	0.463079461661424	1
Rv3804 (Ag85A)	15 (100)	15 (100)	15 (100)	15	15	15	1	1
Rv1886 (Ag85B)	14 (93)	15 (100)	14 (93)	14	15	14	1	1
Rv1917	14 (93)	12 (80)	13 (87)	14	12	13	0.857873975145676	1
Rv0978	13 (87)	14 (93)	13 (87)	13	14	13	0.999999999999999	1
Rv0754	14 (93)	15 (100)	14 (93)	14	15	14	1	1

Patients were recruited from the Tuberculosis Research Center in Belarus. H= Healthy, P=patients, AFS and culture negative, yet previous TB. TB= patients suffering from TB. n=15 in each group. The numbers in each designate a positive response defined by IFN-gamma production in the WBA; the percentage of responders in each group is provided in brackets. The p-values as well as the adjusted p-values (adjusted for the FDR) are provided.
